# Characterization of the complete chloroplast genome of *Quercus acrodonta* (Fagaceae)

**DOI:** 10.1080/23802359.2021.1994893

**Published:** 2021-11-17

**Authors:** Xuan Li, Yongfu Li, Yousry A. El-Kassaby, Yanming Fang

**Affiliations:** a College of Biology and the Environment, Key Laboratory of State Forestry and Grassland Administration on Subtropical Forest Biodiversity Conservation, Co-Innovation Center for Sustainable Forestry in Southern China, Nanjing Forestry University, Nanjing, China; b Department of Forest and Conservation Sciences, Faculty of Forestry, The University of British Columbia, Vancouver, Canada

**Keywords:** *Quercus*, cp genome, *Quercus acrodonta*, Fagaceae

## Abstract

*Quercus acrodonta* Seemen is an East Asian evergreen oak tree species belonging to the *Quercus* section *Ilex*. Here, we assembled and annotated the complete chloroplast (cp) genome of the species. The circular genome is 161,105 bp in size, presenting a typical quadripartite structure including two copies of inverted repeat (IR) regions (25,864 bp), one large single-copy (LSC) region (90,357 bp), and one small single-copy (SSC) region (19,020 bp). A total of 131 genes are encoded, including 85 protein-coding genes (PCGs), 38 tRNAs, and eight rRNAs. Phylogenetic analysis based on cp genome sequences of 18 *Quercus* species indicated that *Q*. *acrodonta* was more closely related to *Q*. *phillyraeoides*.

*Quercus acrodonta* Seemen, is an evergreen oak tree species endemic to China and belonging to the *Quercus* section *Ilex* (Denk et al. 2017). It is widely distributed in Gansu, Guizhou, Henan, Hubei, Sichuan, Shanxi, and Yunnan Provinces. Although a widespread species, it is not common and its populations tend to be small. The species leaf morphology varies with the change of environment, thus increasing the difficulty of taxonomists' work. Few *Q. acrodonta* molecular studies were conducted and mainly aimed at understanding the species distribution and physiology. The conservative nature of the chloroplast (cp) genome (gene content and organization) made it a useful tool for species identification (Szmidt et al. 1988) and resolving phylogenetic relationships (Yang et al. 2016). Here, we assembled and characterized the complete cp genome for *Q. acrodonta* from high-throughput sequencing data and used the cpDNA sequence information along with additional 17 *Quercus* species from NCBI (*Q. baronii*, *Q. dolicholepis*, *Q. acutissima*, *Q. variabilis*, *Q. tarokoensis*, *Q. edithiae*, *Q. glauca*, *Q. sichourensis*, *Q. aquifolioides*, *Q. spinosa*, *Q. tungmaiensis*, *Q. aliena* var*. acutiserrata*, *Q. aliena*, *Q. phillyraeoides*, *Q. chenii*, *Q. dentate*, and *Q. rubra*) to construct a phylogenetic analysis to determine their evolutionary relationship.

Total genomic DNA was isolated from fresh leaves of a single *Q. acrodonta* individual (Hangzhou Botanical Garden, Hangzhou, China; 120°7′28″E, 30°15′35″N) using DNeasy Plant Mini Kit (Qiagen, Valencia, CA), and was used to prepare the shotgun library following the manufacturer’s protocol for Hiseq4000 Sequencing System (Illumina, San Diego, CA). A specimen was deposited at the Herbarium of Nanjing Forestry University (contact Xuan Li and xuanli18851128817@163.com) under the voucher number: LX2020101205. The library was sequenced by Nanjing Genepioneer Biotechnologies Inc. (Nanjing, China). A total of 39,278,722 raw reads were obtained and used for the *de novo* assembly with NOVOplasty 2.7.2 (Dierckxsens et al. 2016). The resultant genome was annotated by CpGAVAS (Liu et al. 2012).

The complete plastid genome of *Q. acrodonta* (GenBank accession number MW553099) is a circular molecule of 161,105 bp in length. It contained two inverted repeats (IRa and IRb) regions of 25,864 bp, which were separated by a large single-copy (LSC) region of 90,357 bp and a small single-copy (SSC) region of 19,020 bp. A total of 131 genes are encoded, including 85 protein-coding genes (PCGs), 38 tRNAs, and eight rRNAs. Among them, we found six PCGs, four rRNAs, and seven tRNA genes were duplicated. The overall GC content of the *Q. acrodonta* genome is 36.8% and the corresponding values in LSC, SSC, and IR regions are 34.6, 30.9, and 42.7%, respectively.

A maximum-likelihood (ML) tree of *Quercus* species was reconstructed to identify the phylogenetic position of *Q. acrodonta* and *Ulmus gaussenii* W.C. Cheng (GenBank: NC_037840) was selected and used as an outgroup species. Maximum-likelihood analyses were conducted using complete cp genome data in IQ-TREE v 1.6.12 (Nguyen et al. 2015) under the GTR substitution model with 10,000 Ultrafast bootstrap analyses along with a search for the best-scoring tree in 1000 iterations. The results indicated that *Q. acrodonta* belonged to the *Ilex* section, and was most closely related to *Q. phillyraeoides* with 80% bootstrap support. Furthermore, we confirmed that the section *Ilex* is not a monophyletic group based on the complete cp genome (Figure 1).

**Figure 1.**
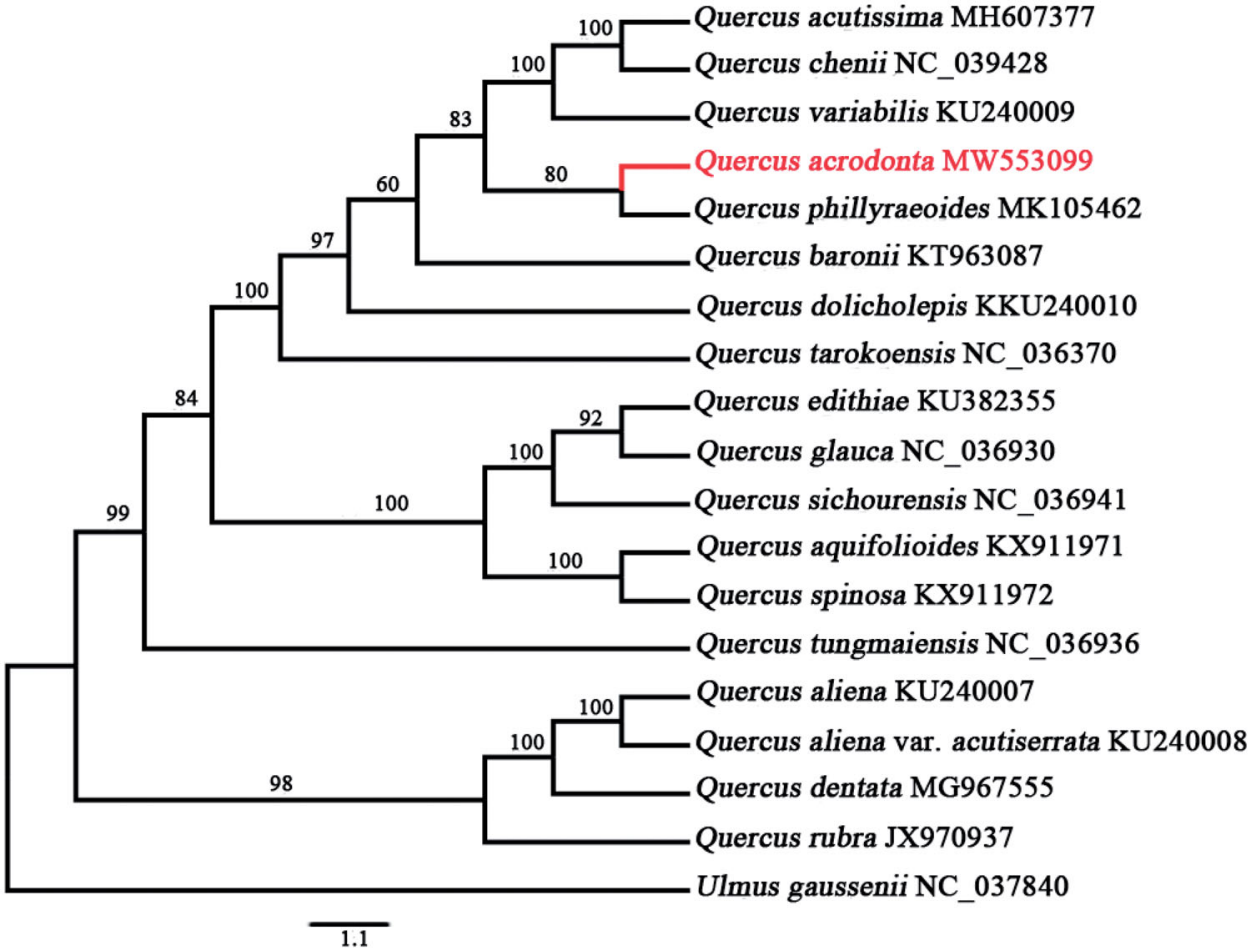
Maximum-likelihood phylogenetic relationship among 18 complete chloroplast genomes of *Quercus* species. Bootstrap support values are labeled at each node. *Ulmus gaussenii* was used as an outgroup. Sequence data source is listed after each species name.

## Data Availability

The genome sequence data that support the findings of this study are openly available in GenBank of NCBI at https://www.ncbi.nlm.nih.gov under the accession MW553099.
